# Medium-term impact of the economic crisis on mortality, health-related behaviours and access to healthcare in Greece

**DOI:** 10.1038/srep46423

**Published:** 2017-04-10

**Authors:** Filippos T. Filippidis, Vasiliki Gerovasili, Christopher Millett, Yannis Tountas

**Affiliations:** 1 Department of Primary Care and Public Health, School of Public Health, Imperial College London, United Kingdom; 2 Center for Health Services Research, School of Medicine, National and Kapodistrian University of Athens, Athens, Greece; 3 Department of Respiratory Medicine, Harefield Hospital, Royal Brompton & Harefield NHS Foundation Trust, London, United Kingdom

## Abstract

Previous studies on the health consequences of the crisis in Greece investigated short-term impacts on selected outcomes. This study examined the impact of the crisis on a key set of health indicators with longer follow up than previous studies. We conducted interrupted time series (ITS) analysis to compare trends in standardised mortality by cause before and during the crisis. We examined changes in fruit and vegetable consumption, smoking, physical activity, obesity, out-of-pocket payments and unmet needs for healthcare using national household data from the “Hellas Health” surveys. Standardised mortality rates for suicides (p < 0.001) and infant mortality (p = 0.003) increased during the crisis compared to pre-existing trends, while mortality from respiratory diseases (p = 0.053) and transport accidents (p = 0.067) decreased. The prevalence of smoking (42.6% to 36.5%; RR = 0.86) and sedentary lifestyle (43.4% to 29.0%; RR = 0.69) declined. The prevalence of unmet need for healthcare significantly increased from 10.0% to 21.9% (RR = 2.10) and the proportion of people paying out-of-pocket for healthcare from 34.4% to 58.7% (RR = 1.69) between 2010 and 2015. The impact of the economic crisis in Greece on health was more nuanced than previous reports suggest. Effective strategies to mitigate the adverse health impacts of economic crises need to be better understood and implemented.

Greece has been hit hardest by the recent economic crisis in Europe. While the recession started in 2008, it was not until 2010, when severe austerity measures were imposed in the country^1^,^2^, that its consequences were felt in Greek society. Until early 2010, the rise in unemployment was minimal and the wages were increasing^3^. Between 2009 and 2014, expenditure on healthcare decreased by more than 25%, average wages decreased by 20% and unemployment increased from 9.6% to 26.5%^3^,^4^. The proportion of the population at-risk-of-poverty and suffering from material deprivation increased sharply within the same period (16.4% to 42.7% and 23.0% to 49.5% respectively)^3^,^5^. (Supplementary Table S1).

Previous studies suggest that the crisis has resulted in increases in poor self-rated health^6^, depression^7^, suicide attempts^8^, homicides^9^, infant^10^ and all-cause mortality^11^, outbreaks of infectious diseases^12^, restrictions in access to healthcare^2^,^13^, and mortality due to adverse events during medical treatment^14^. However, most of these studies included data only from the first two years of the crisis and only report short-term effects. As a result, there is still a lot of uncertainty regarding the health effects of the crisis in Greece, which reflects a controversial evidence base on the short- and long-term impact of economic downturns on health in general^15^.

The case of Greece may be relevant to other countries, which are also affected by global economic downturn and are faced with choices about whether to adopt austerity measures. Thus, the aim of the current study was to examine the medium-term impact of the economic crisis on a comprehensive set of health indicators including mortality; mental health; behavioural risk factors; and access to healthcare, using the most recent available data.

## Results

### Mortality

**Figure 1:**
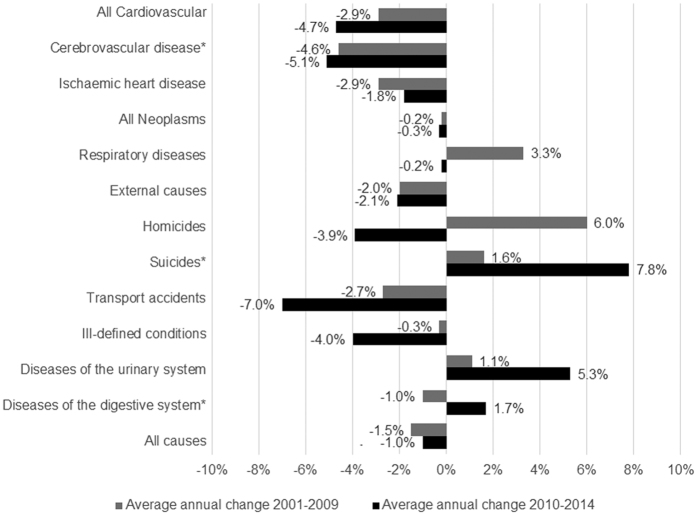
Average annual changes in standardised mortality (per 100,000) by selected causes of death in Greece, before and during the crisis. *The star indicates a statistically significant (p < 0.05) change in slopes before and during the crisis, based on the interrupted time series analysis. Average annual change of standardised mortality from all causes during the crisis was calculated for years 2010 to 2015. Respiratory diseases include ICD-9 codes 460–519 and ICD-10 codes J00-J98; all neoplasms include ICD-9 codes 140-239 and ICD-10 codes C00-D48; cardiovascular diseases include ICD-9 codes 390–459 and ICD-10 codes I00-I99; ischaemic heart disease include ICD-9 codes 410–414 and ICD-10 codes I20–I25; cerebrovascular disease include ICD-9 codes 430–438 and ICD-10 codes I60–I69; hypertensive disease include ICD-9 codes 401–405 and ICD-10 codes I10–I15; external causes include ICD-9 codes e800-e999 and ICD-10 codes V01-Y89; transport accidents include ICD-9 codes E800-E848 and ICD-10 codes V01-V99, Y85; suicides and self-inflicted injury include ICD-9 codes E950-E959 and ICD-10 codes X64-X84, Y870; homicide and injury purposely inflicted by other persons include ICD-9 codes E960-E969 and ICD-10 codes X85-Y09, Y871; signs, symptoms and ill- defined conditions include ICD-9 codes 780–799 and ICD-10 codes R00–R99; diseases of the digestive system include ICD-9 codes 520–579 and ICD-10 codes K00–K92; and diseases of the urinary system include ICD-9 codes 580–629 and ICD-10 codes N00–N98.

Standardised all-cause mortality was declining by an average 1.5% per year before the crisis and kept declining by an average 1.0% per year between 2010 and 2015 (p-value of the Interrupted Time Series [ITS] analysis = 0.986), despite an increase of 3.4% in 2015, compared to the previous year (Fig. 1, Supplementary Table S2). Standardised mortality from the two main causes of death in Greece, cardiovascular disease and neoplasms, continued to fall during the crisis, while mortality from respiratory diseases declined during the crisis, in contrast to its increasing trend before 2009 (p = 0.053). Similarly, the average annual decline in mortality from transport accidents was 7.0% during the crisis, compared to just 2.7% between 2001 and 2009 (p = 0.067). On the contrary, standardised mortality from suicides increased by an average 7.8% per year after 2009, compared to 1.6% before the economic crisis (p = 0.001). Despite variation in total mortality between geographic regions, mortality trends were quite similar in all regions after 2009 (Supplementary Table S3). Declines in all-cause mortality during the crisis were greatest in persons below 50 years of age (Supplementary Table S4).

**Figure 2:**
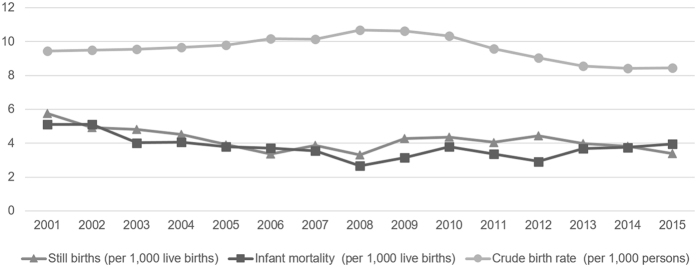
Crude birth rate, still births and infant mortality in Greece, 2001–2015.

Between 2010 and 2015, the crude birth rate in Greece declined by an average 3.9% per year (p-value of the ITS analysis <0.001). Still births decreased at an annual rate of 4.6%, continuing the declining trend of the past decades (p = 0.380), but infant mortality increased by 1.8% per year compared to an average annual decline of 2.1% between 2001 and 2009 (p = 0.003). The proportion of live births classified as low birth weight remained stable (9.6% in 2009; 9.3% in 2015) (Fig. 2).

### Self-reported mental health and health-related quality of life

**Figure 3:**
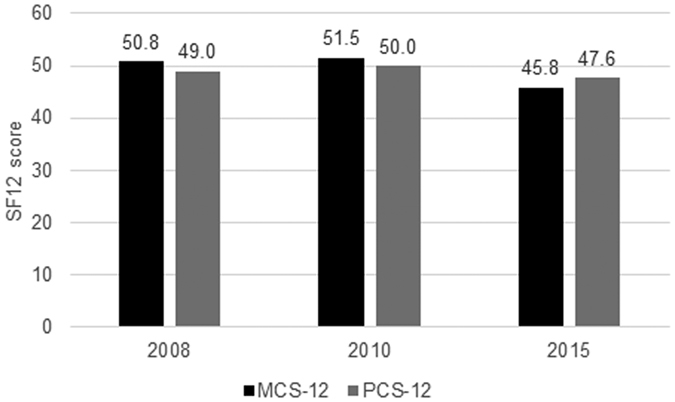
Health-related quality of life in Greece, 2008–2015. MCS-12: Mental composite score of SF12; PCS-12: Physical composite score of SF12.

The prevalence of diagnosed mental health problems did not differ between 2010 and 2015 (6.9% and 6.3% respectively; Risk Ratio [RR] = 0.87; 95%Confidence Interval [CI]: 0.61 to 1.23). However, we found a statistically significant decline in both health-related quality of life composite scores between 2008 and 2015. MCS-12 decreased by 5.2 (95% CI: −6.6 to −3.7) and PCS-12 by 1.8 points (95% CI: −2.7 to −1.0) (Fig. 3).

### Risk factors for chronic diseases

Between 2008 and 2015, the prevalence of smoking among adults decreased from 42.6% to 36.5% (RR = 0.86; 95% CI: 0.77 to 0.95), while sedentary lifestyle decreased from 43.4% to 29.0% between 2006 and 2015 (RR = 0.69; 95% CI: 0.61 to 0.79). Fruit and vegetable consumption (RR = 1.00; 95% CI: 0.92 to 1.09), as well as the prevalence of obesity (RR = 0.98; 95% CI: 0.81 to 1.19) didn’t change significantly during the crisis (Table 1).

### Healthcare

The proportion of Greek adults reporting unmet healthcare needs was 10.0% in 2010 and 21.9% in 2015 (RR = 2.10; 95% CI: 1.66 to 2.64). The proportion of the respondents who cited cost as a reason significantly increased from 3.4% to 12.2%, and of those who reported accessibility issues from 2.8% to 4.5% during the same period. Any out of pocket payments in the past 12 months were reported by 34.4% of the respondents in 2010 and by 58.7% in 2015 (RR = 1.69; 95% CI: 1.52 to 1.89) (Table 2), with the mean Out-of-Pocket Expenditure (OOPE) in 2015 being €296.1 compared to €147.5 in 2010. Among those who reported any OOPE, the mean amount spent in the past 12 months was not significantly different between the two surveys. Finally, the proportion of respondents with private health insurance was 8.4% in 2008, dropped to 5.8% in 2011 and increased to 12.4% in 2015. The difference was significant between 2011 and 2015, but not between the 2008 and the most recent wave.

## Discussion

We found that during the ongoing economic crisis in Greece overall mortality continued to decline; however data from the most recent year (2015) showed an increase in all-cause mortality. Suicides and infant mortality have increased, but standardised mortality from respiratory diseases and transport accidents has declined. The prevalence of smoking and physical inactivity also declined, but health-related quality of life scores, unmet healthcare needs and OOPE worsened significantly over the same period.

One issue that has already been highlighted as a negative consequence of the economic crisis is the increase in suicides and suicide attempts^15^,^16^. Our analysis showed that the standardised mortality from suicides increased rapidly after 2009. However, Greece still has one of the lowest suicide mortality rates among developed countries^17^; therefore the impact of this increase on all-cause mortality is very limited. In 2014, there were 174 more suicides compared to 2009 in the entire country. Nevertheless, suicide rates may reflect worsening mental health at a population level, as well as inadequate mental health services^18^. Indeed, depressive symptoms among the general population have increased during the crisis^7^ and the average score of the SF-12 mental health component declined sharply, indicating deteriorating mental health in the country. The proportion of adults diagnosed by a physician with either depression or anxiety disorders did not increase in our study, but this may imply that these problems are being under-diagnosed, as mental health services are under a lot of pressure and understaffed^19^. The mix of poor access to mental health services, untreated mental disorders, unemployment and financial loss, which are among the most important risk factors for suicide, is contributing to the aggravation of the suicide problem in Greece.

Despite poor enforcement of tobacco control measures by successive Greek governments^20^, we found that there were 540,000 fewer adult smokers in 2015 compared to 2008 while the annual cigarette stick consumption per capita dropped from 3,164 to 1,979 sticks in just five years^21^. Reduced disposable income has been the main driver of this decline^22^ which can lead to significant short- and long-term health gains. The sharp decline in mortality from respiratory diseases observed until 2013 could be interpreted as an early sign of such gains^14^,^23^, but we found that standardised respiratory mortality in 2014 was higher than the previous year, indicating that factors influencing respiratory mortality may be complex^23^.

Similarly, increased physical activity levels are greatly beneficial for the population’s health, especially as they seem to be associated with less usage of private cars^24^, which has led to a reduction in the number of road traffic accidents victims. Less reliance on private vehicles has also contributed to lower emissions and cleaner air in urban areas, such as Athens^25^, even though this is partly being undermined by the increasing popularity of wood combustion for heating^5^.

Barriers to healthcare access emerged as one of the main negative consequences of austerity, as the proportion of Greek adults who reported unmet healthcare needs more than doubled in just five years, with 12.2% of the respondents citing cost as the main reason for not receiving treatment or diagnostic tests. The Greek healthcare system suffered major spending cuts, but the underlying inefficiencies were not effectively addressed^2^,^26^, forcing large numbers of health professionals out of the country^27^. Moreover, the introduction of user fees and co-payments shifted some of the cost to patients adding further barriers to timely healthcare access^2^,^13^. Our survey data showed that average OOPE almost doubled between 2010 and 2015, mostly due to greater number of people being required to pay for prescription drugs^28^ and healthcare services, either as user fees for public services or increasing use of private services to avoid deteriorating conditions in the public healthcare system. Other European countries experienced increases in OOPE, following the recent recession^29^. The impact of the crisis on healthcare extends beyond access factors and may include declining quality of care, aging infrastructure and staff shortages, which might have important short- and long-term implications for morbidity and mortality, such as adverse events of medical care, which seem to have increased in Greece during the crisis^14^,^30^.

Despite earlier reports of increasing homicides^9^, and all-cause mortality^11^, our comparison with pre-crisis trends revealed no evidence of changing trends during the crisis, which is in line with mortality trends observed during recessions in the past^15^. Close monitoring is required in order to explore whether the increase observed in 2015 signifies a dramatic change in the course of the Greek crisis or it was a statistical anomaly without further implications. Infant mortality has increased by 26% during the crisis, potentially indicating persisting problems in perinatal care and access to essential health services. Overall, younger people seem to have benefited more from changes in mortality; there was little or no improvement in mortality among those aged 50–69 years, who may already suffer from chronic diseases and are more reliant to the healthcare system.

We found no evidence of differential changes in health-related quality of life, mental health, risk factors and unmet needs by SES or employment status, in contrast to an earlier study which reported a growing health inequality gap^31^. This could be explained by the relatively small sample size. Nevertheless, it might, at least in part, have to do with the particular characteristics of the Greek society. Informal support networks, especially within families, are still quite strong in Greece. As a result, conventional measures of SES, such as occupation and education may not reflect the actual financial and social circumstances of individuals. Other indicators of SES, for example persons living in households with very low work intensity, which increased rapidly during the economic crisis, could have identified differential impacts by socio-economic position.

### Strengths and limitations

To the authors’ knowledge, this is the first study to explore medium-term trends in such a comprehensive set of health-related indicators and standardised mortality in Greece during the economic crisis. We used official mortality data, as well as data collected through a series of cross-sectional surveys that cover a period long enough to detect trends over time, adjusting for potential confounding factors. However, we were not able to analyse a longitudinal sample, therefore causal interpretation of our findings should be made with caution. Data on certain indicators were not collected in some survey waves, in which case we made comparisons using data from the closest available year. Additionally, we could not fully explore potential social inequalities and how the crisis has influenced them. The coding of cause-specific mortality changed from ICD-9 to ICD-10 between 2013 and 2014, but this did not affect our analyses, as we used broad disease groups, which do not differ between the two classifications. Finally, we used either aggregate data, which could mask individual-level effects, or self-reported data on individuals, which are subject to information bias. Future research could focus on cohort studies with objective measures of smoking and diet.

## Conclusion

Our findings show that there are some areas, such as smoking, physical activity, respiratory diseases and traffic accidents, in which trends have been favourable during the economic crisis, and others, such as infant mortality, mental health and access to healthcare, in which conditions have significantly worsened. Identifying and implementing effective strategies to mitigate the adverse health impacts of economic crises is an important priority for scientists and politicians alike.

## Methods

### Data sources

#### Hellenic Statistical Authority

The number and causes of deaths and estimated population by sex and 5-year age groups for years 2001 to 2015, as well as data on birth outcomes were downloaded from the official Hellenic Statistical Authority’s (ELSTAT) website^3^, both for the entire country and for each of its thirteen regions.

#### Hellas Health Surveys

We analysed data from five waves of the Greek national household “Hellas Health” (HH) surveys. The five waves of the survey were conducted in October 2006 (n = 1,005), June 2008 (n = 1,490), October 2010 (n = 1,000), October 2011 (n = 1,008), and April 2015 (n = 1,001). A three stage sampling design based on the 2001 population census was followed (the 2011 census was used for the 2015 wave). At the first stage, a random sample of building blocks was selected proportionally to size, covering both urban (2,000 or more inhabitants) and rural areas (less than 2,000 inhabitants) of the country and each of the 13 geographical regions. At the second stage, in each selected area of blocks, systematic sampling was used to randomly select the households to be interviewed. Any person or group of persons living in a separate housing unit was considered as a “household” unit. At the third stage, in each household, a sample of individuals aged >18 years old was selected by means of simple random sampling. Data were collected through interviews with respondents aged ≥18 years. Samples in each wave were independently selected. Response rates ranged from 44.1% to 51.0% in all surveys and resulted in samples representative of the Greek adult population in terms of gender, age and residency (Supplementary Table S5).

A core questionnaire assessing risk factors and sociodemographic data was used in all waves, and additional modules appeared in some of the waves. More details on the sampling methodology and survey instruments have been reported elsewhere^24^. Ethics approval for the Hellas Health surveys has been provided by the ethical committee of the National and Kapodistrian University of Athens, Faculty of Medicine. Informed consent was obtained from all respondents and data collection and analysis were conducted in accordance to relevant guidelines and regulations.

### Measures

#### Mortality

ELSTAT reported mortality by 56 causes of death, based on version 9 of the International Classification of Diseases (ICD-9) until 2013 and on ICD-10 in 2014^3^. Mortality data by cause were not available for year 2015 at the time of the analysis.

#### Birth outcomes

Low birth weight was defined as birth weight lower than 2,500 grams. Crude birth rate was calculated as the number of births per 1,000 persons. Infant mortality and stillbirths are presented per 1,000 live births and per 1,000 live and still births respectively.

#### Self-reported mental health

HH respondents who reported having been diagnosed by a doctor with anxiety disorder or/and depression were classified as having a mental health problem.

#### Health-related quality of life

Health-related quality of life in HH was assessed with the Short Form 12 Health Survey version 1 (SF-12v1); standardised Mental (MCS-12) and Physical Composite Scores (PCS-12) were calculated for each individual. Higher composite scores (possible range: 0 to 100) indicate better quality of life.

#### Risk factors for chronic diseases

HH participants self-reported the number of fruit and vegetable servings they consume daily; their smoking status; and their height and weight. Body Mass Index (BMI) was calculated using self-reported data for height and weight and those with BMI ≥30 kg/m^2^ were classified as obese. Self-reported daily intake of ≤2 servings of fruit and vegetables was considered as low consumption^32^. Levels of physical activity were assessed with the International Physical Activity Questionnaire (IPAQ)^33^, which classifies respondents into three levels of physical activity; those in the lower activity level were classified as sedentary.

#### Healthcare

HH respondents were asked “during the past 12 months, was there any time when, in your opinion, you needed treatment or diagnostic test for a health problem, but you didn’t receive it?”. Positive responses indicated unmet healthcare need. Those who reported unmet healthcare need were also asked to identify up to two reasons, including cost (“I could not pay for treatment/tests”); access (“long distance from doctor/health service”; “I couldn’t go during the doctor’s/health service’s opening times”; and “long waiting time for an appointment”); and others, such as “lack of trust”, “being busy”, and “not knowing where to go”.

Out of pocket expenditure was also assessed for the past 12 months, as respondents were asked to report the total amount of money (in Euros) they paid out of pocket for diagnostic tests, treatments and visits to health professionals as outpatients, thus excluding hospital stays. Finally, they were asked to report whether they have a private health insurance.

#### Sociodemographic

HH respondents were asked to report their gender, age (18–34; 35–54; and >54 years), level of education (no education/primary; secondary; and higher); area of residence (urban; rural) and occupation (employed; unemployed; not working). Respondents were classified into four groups of socioeconomic status (SES), according to the ESOMAR scale (high = A/B; upper middle = C1; lower middle = C2; low = D/E). The ESOMAR scale assigns a socioeconomic level to a participant according to the occupation and education of the main income earner of the respective household^34^.

### Statistical analysis

We employed direct standardisation methods (by sex and 5-year age groups) to calculate disease- and region-specific standardised mortality rates, using the population of Greece in 2009 as the standard population. The average annual change in mortality before the crisis was calculated as the mean of annual changes between 2001 and 2009 (2002–2009 for region-specific mortality) and the average annual change during the crisis was calculated as the mean of annual changes between 2010 and 2014. We conducted an interrupted time series analysis for all mortality and birth outcomes, with 2010 as the year of the “intervention”. While ITS typically report level (immediate) and slope (gradual) changes in outcome measures we only report the latter, as immediate changes in mortality levels following the introduction of austerity measures are unlikely^14^,^35^.

Differences between earlier waves –depending on availability of data- and the most recent (2015) wave of the Hellas Health survey were assessed with logistic –for categorical variables- or linear –for continuous variables- regression models, adjusted for age; gender; area of residence; education; occupation; and SES. Interaction terms between wave and SES, as well as between wave and occupation were included in the models, but were removed from the final models, as they were not statistically significant at the 0.05 level in any of the analyses. Logistic regression results are presented as Risk Ratios (RR) with 95% Confidence Intervals (CI) and linear regression results as β coefficients with 95% CI. All analyses were conducted using Stata 14.0 (StataCorp LP, College Station, TX).

### Ethics approval

Ethics approval for the Hellas Health surveys has been provided by the ethical committee of the National and Kapodistrian University of Athens, Faculty of Medicine.

### Availability of data and material

All data from the Hellenic Statistical Authority are publicly available on their webpage www.statistics.gr. The HH datasets generated and/or analysed during the current study are available from the corresponding author on reasonable request.

## Additional Information

**How to cite this article:** Filippidis, F. T. *et al*. Medium-term impact of the economic crisis on mortality, health-related behaviours and access to healthcare in Greece. *Sci. Rep.* **7**, 46423; doi: 10.1038/srep46423 (2017).

**Publisher's note:** Springer Nature remains neutral with regard to jurisdictional claims in published maps and institutional affiliations.

## Supplementary Material

Supplementary Tables

## Figures and Tables

**Table 1:** Risk factors in Greece, 2008–2015.

	2008 % (95% CI)	2010 % (95% CI)	2011 % (95% CI)	2015 % (95% CI)	RR^a^ (95% CI) 2015 vs. pre-crisis^b^
Smoking	42.6 (40.0 to 45.1)	42.6 (39.6 to 45.7)	37.9 (34.9 to 40.9)	36.5 (33.3 to 39.7)	0.86 (0.77 to 0.95)
Sedentary lifestyle	43.4 (40.2 to 46.6)^c^	—	31.8 (28.9 to 34.7)	29.0 (26.0 to 32.0)	0.69 (0.61 to 0.79)
Low fruit/vegetable consumption	52.1 (49.6 to 54.7)	—	51.3 (48.2 to 54.4)	51.2 (47.9 to 54.6)	1.00 (0.92 to 1.09)
Obesity	18.1 (16.1 to 20.1)	18.2 (15.8 to 20.6)	17.5 (15.1 to 19.9)	17.4 (14.9 to 19.9)	0.98 (0.81 to 1.19)

^a^Risk Ratio (RR) adjusted for age, gender, area of residence, education, occupation and SES.

^b^Pre-crisis means the 2008 survey, or 2006 if data not available for 2008.

^c^Data from 2006.

**Table 2:** Unmet healthcare needs, private insurance and out of pocket expenditure in Greece, 2010–2015.

	2010% or mean (95% CI)	2011% or mean (95% CI)	2015% or mean (95% CI)	2015 vs. 2010 RR^a^ or β^b^ (95% CI)
Unmet healthcare need (any reason)	10.0 (8.0 to 11.8)	—	21.9 (19.2 to 24.6)	2.10 (1.66 to 2.64)
Unmet healthcare need (cost)	3.4 (2.3 to 4.5)	—	12.2 (10.1 to 14.4)	3.31 (2.24 to 4.87)
Unmet healthcare need (access)	2.8 (1.8 to 3.8)	—	4.5 (3.1 to 5.9)	1.65 (1.01 to 2.70)
Private insurance	8.4 (6.4 to 10.3)^c^	5.8 (3.8 to 7.8)	12.4 (9.8 to14.9)	1.28 (0.94 to 1.74)
Any out of pocket expenditure	34.4 (31.4 to 37.4)	—	58.7 (55.1 to 62.3)	1.69 (1.52 to 1.89)
Out of pocket expenditure in € (entire sample)	147.5 (108.4 to 186.6)	—	296.1 (251.6 to 340.5)	+137.2 (77.0 to 197.4)
Out of pocket expenditure in € (among those who reported any OOPE)	428.7 (321.2 to 536.1)	—	504.6 (435.5 to 573.6)	+77.4 (−52.2 to 207.0)

^a^Risk Ratio (RR) for categorical variables adjusted for age, gender, area of residence, education, occupation and SES.

^b^β coefficient for continuous variables adjusted for age, gender, area of residence, education, occupation and SES.

^c^Data from 2008; not available for 2010.
