# Supplementary material for: Medium-term impact of the economic crisis on mortality, health-related behaviours and access to healthcare in Greece
Source: Sci Rep. 2017 Apr 10;7:46423. doi: 10.1038/srep46423 (PMC5385490; doi:10.1038/srep46423)
Supplement: Supplementary Tables [file srep46423-s1.pdf]

Medium-term impact of the economic crisis on mortality, health-related behaviours and access to healthcare in Greece

Filippos T. Filippidis[1,2], Vasiliki Gerovasili[3], Christopher Millett[1], Yannis Tountas[2]

[1] Department of Primary Care and Public Health, School of Public Health, Imperial College London, United Kingdom

[2] Center for Health Services Research, School of Medicine, National and Kapodistrian University of Athens, Athens, Greece

[3] Department of Respiratory Medicine, Harefield Hospital, Royal Brompton & Harefield NHS Foundation Trust, London, United Kingdom

Supplementary Table S1. Macroeconomic indicators in Greece, 2009-2014

	2009	2010	2011	2012	2013	2014	Change 2009-2014
GDP per capita (€)	21,386	20,324	18,643	17,311	16,451	16,250	-25.6%
Government expenditure on healthcare (US$ PPP)	2,971	2,645	2,598	2,319	2,357	2,098	-29.4%
Unemployment	9.6%	12.7%	17.9%	24.4%	27.5%	26.5%	+176.0%
Gini coefficient	33.1	32.9	33.6	34.3	34.4	34.5	+4.2%
Seasonally adjusted Wage Index	113.0	111.4	106.2	96.1	88.8	90.4	-20.0%
At-risk-of-poverty after social transfers [a]	19.7%	20.1%	21.4%	23.1%	23.1%	22.1%	+12.2%
At-risk-of-poverty anchored at a fixed moment in year 2005 [b]	16.4%	16.3%	22.9%	32.3%	40.0%	42.7%	+160.4%
Living in households with very low work intensity [c]	6.5%	7.5%	11.8%	16.1%	19.6%	19.4%	+198.5%
Material deprivation [d]	23.0%	24.1%	28.4%	33.7%	37.3%	39.5%	+71.7%
Households by main heating means							
Central heating	73.5%	73.1%	72.1%	63.8%	47.0%	44.7%	-39.2%
Firewood stove	5.9%	5.4%	6.7%	7.9%	11.6%	11.0%	+86.4%

[a] defined as the proportion of the population living in households where the total equivalised disposable income is lower than 60% of the national median equivalised disposable income.

[b] defined as the share of the population whose total equivalised disposable income is lower than 60% of the national median equivalised disposable income of 2005, adjusted for inflation.

[c] defined as the proportion of the population aged 18-59 years living in a household where household members of working age worked <20% of their total work potential over the past year.

[d] defined as the proportion of the population that cannot afford at least three of the following items: to pay arrears on mortgage or rent payments, or utility bills, hire purchase instalments or other loan payments; to go on one week's annual holiday away from home; to have a meal with meat, chicken,

fish (or vegetarian equivalent) every second day; to face unexpected expenses; to have a telephone (including mobile phone); to have a colour TV; to have a washing machine; to have a car; or to keep their home adequately warm.

Supplementary Table S2. Standardised mortality (per 100,000) by selected causes of death in Greece, 2001-2014.

	2001	2002	2003	2004	2005	2006	2007	2008	2009	2010	2011	2012
**Respiratory diseases**	77.5	82.8	79.4	80.4	84.6	88.9	99.6	97.3	99.4	92.4	89.2	93.9
**All Neoplasms**	251.3	250.0	250.5	253.0	253.1	246.4	251.9	251.9	246.5	239.9	236.5	239.3
**All Cardiovascular**	599.7	589.7	591.7	565.8	538.8	532.5	513.5	492.4	471.4	449.9	435.4	434.9
**Ischaemic heart disease**	177.4	176.4	185.8	180.7	162.3	160.5	152.1	138.9	139.4	129.5	128.3	128.1
**Cerebrovascular disease**	248.0	241.7	236.0	221.5	211.7	204.0	188.5	181.5	170.0	158.2	154.1	155.5
**External causes**	41.2	37.6	37.8	38.6	39.1	36.9	37.7	34.9	34.9	31.9	30.9	30.9
**Transport accidents**	18.6	17.2	16.4	18.2	17.8	16.7	16.2	15.5	14.8	12.9	12.1	10.8
**Suicides and self-inflicted injury**	3.7	3.5	4.0	3.8	4.3	4.2	3.4	3.9	4.0	3.8	4.8	5.1
**Homicide and injury purposely inflicted by other persons**	1.4	1.0	1.4	1.2	1.3	1.2	1.5	1.8	1.9	1.9	2.2	2.0
**Signs, symptoms and ill- defined conditions**	124.2	132.0	140.1	139.4	138.2	114.8	128.6	109.0	116.4	143.8	162.5	167.1
**Diseases of the digestive system**	31.9	32.7	31.7	29.9	30.4	31.1	30.2	29.4	29.3	28.5	30.0	30.7
**Diseases of the urinary system**	21.5	18.8	18.3	18.3	18.7	22.4	27.5	27.0	21.9	21.6	19.6	19.9
**All causes**	1086.3	1083.1	1084.1	1063.1	1043.4	1022.1	1037.6	997.9	976.3	956.4	946.0	959.1

Mortality rates have been standardised by gender and 5-year age groups, using the population of Greece in 2009 as the standard population.

Respiratory diseases include ICD-9 codes 460-519 and ICD-10 codes J00-J98; all neoplasms include ICD-9 codes 140-239 and ICD-10 codes C00-D48; cardiovascular diseases include ICD-9 codes 390-459 and ICD-10 codes I00-I99; ischaemic heart disease include ICD-9 codes 410-414 and ICD-10 codes I20-I25; cerebrovascular disease include ICD-9 codes 430-438 and ICD-10 codes I60-I69; hypertensive disease include ICD-9 codes 401-405 and ICD-10 codes I10-I15; external causes include ICD-9 codes e800-e999 and ICD-10 codes V01-Y89; transport accidents include ICD-9 codes E800-E848 and ICD-10 codes V01-V99, Y85; suicides and self-inflicted injury include ICD-9 codes E950-E959 and ICD-10 codes X64-X84, Y870; homicide and injury purposely inflicted by other persons include ICD-9 codes E960-E969 and ICD-10 codes X85-Y09, Y871; signs, symptoms and ill- defined conditions include ICD-9 codes 780-799 and ICD-10 codes R00-R99; diseases of the digestive system include ICD-9 codes 520-579 and ICD-10 codes K00-K92; and diseases of the urinary system include ICD-9 codes 580-629 and ICD-10 codes N00-N98.

Supplementary Table S3. Standardised mortality (per 100,000) by geographic region in Greece, 2002-2014.

	2002	2003	2004	2005	2006	2007	2008	2009	2010	2011	2012	2013
**Eastern Macedonia and Thrace**	1196	1202	1167	1175	1148	1152	1095	1087	1103	1047	1051	1007
**Central Macedonia**	1168	1170	1143	1127	1100	1107	1067	1030	1022	977	1004	926
**Western Macedonia**	1053	1076	1062	1031	1007	1000	1008	968	961	937	951	901
**Thessaly**	1115	1051	1086	1007	986	1037	996	991	950	958	960	884
**Epirus**	942	931	923	909	898	895	906	845	797	825	824	798
**Ionian Islands**	1073	1060	1031	977	1019	976	1022	972	889	875	930	876
**Western Greece**	980	1032	1018	1032	1008	1016	991	981	961	945	964	908
**Central Greece**	1001	1006	1006	971	963	1002	957	902	883	926	937	834
**Peloponnese**	948	953	934	920	944	955	934	928	902	895	916	835
**Attica**	1146	1138	1108	1082	1036	1059	987	986	964	959	965	918
**North Aegean**	991	1037	999	972	981	979	923	943	906	867	936	851
**South Aegean**	994	1017	946	965	923	960	894	914	897	929	899	845
**Crete**	982	1003	948	968	971	972	932	891	893	894	896	833

Mortality rates have been standardised for gender and 5-year age groups, using the population of Greece in 2009 as the standard population.

Supplementary Table S4. Mortality by age group (per 100,000) in Greece, 2002-2015.

Age (in years)	2002	2003	2004	2005	2006	2007	2008	2009	2010	2011	2012	2013
**0 to 4**	115	92	98	93	90	89	73	84	93	78	67	74
**5 to 9**	13	9	14	16	11	12	8	11	11	10	10	10
**10 to 14**	11	13	13	15	11	11	10	13	11	14	9	11
**15 to 19**	42	47	42	40	40	38	32	36	39	33	32	23
**20 to 24**	65	62	69	74	65	68	56	67	57	57	52	43
**25 to 29**	64	62	72	77	69	80	70	73	64	56	52	45
**30 to 34**	70	72	75	73	71	75	73	84	74	68	70	62
**35 to 39**	99	99	98	104	100	98	86	89	89	86	86	77
**40 to 44**	155	156	147	146	144	158	142	144	137	133	132	122
**45 to 49**	251	245	246	235	234	246	232	237	219	226	219	218
**50 to 54**	370	376	382	384	389	378	359	361	356	357	354	346
**55 to 59**	594	605	603	582	563	559	548	554	553	566	573	557
**60 to 64**	851	839	843	815	827	856	851	817	815	791	807	796
**65 to 69**	1413	1404	1368	1285	1258	1264	1186	1168	1143	1202	1257	1229
**70 to 74**	2544	2425	2369	2291	2189	2228	2084	1991	1891	1861	1896	1788
**75 to 79**	4532	4526	4388	4295	4060	4083	3832	3692	3557	3471	3511	3273
**80 to 84**	7958	8008	7796	7728	7515	7637	7445	7274	7001	6826	7032	6408
**85+**	16070	16475	16164	15988	16198	16473	16005	15868	16072	16041	16142	14975
**TOTAL**	1083	1084	1063	1043	1022	1038	998	976	956	946	959	897

Supplementary Table S5. Comparison between survey samples and census data.

		Hellas Health 2006	Hellas Health 2008	Hellas Health 2010	Hellas Health 2011	Hellas Health 2015	Census 2001	Census 2011
Gender								
	Male	48.1%	47.6%	50.6%	48.0%	48.0%	49.5%	49.0%
	Female	52.0%	52.4%	49.4%	52.0%	52.0%	50.5%	51.0%
Age (years)								
	18-34	29.9%	29.6%	28.4%	28.0%	26.9%	32.3%	27.0%
	35-54	32.9%	34.7%	37.2%	36.5%	35.0%	33.4%	35.0%
	≥55	37.2%	35.7%	34.4%	35.5%	38.1%	34.2%	38.0%
Residence								
	Urban	74.6%	69.5%	72.1%	72.4%	77.5%	72.2%	74.5%
	Rural	25.6%	30.5%	27.9%	27.6%	22.5%	27.8%	25.5%
